# Point-of-care platelet function assays demonstrate reduced responsiveness to clopidogrel, but not aspirin, in patients with Drug-Eluting Stent Thrombosis whilst on dual antiplatelet therapy

**DOI:** 10.1186/1477-9560-6-1

**Published:** 2008-02-29

**Authors:** Alex R Hobson, Graham Petley, Geraint Morton, Keith D Dawkins, Nick P Curzen

**Affiliations:** 1 Wessex Cardiac Unit, Southampton University Hospital, Southampton, UK; 2 Department of Medical Physics & Bioengineering, Southampton University Hospitals NHS Trust, Southampton, UK; 3 Southampton University Medical School, Southampton, UK

## Abstract

**Background:**

To test the hypothesis that point-of-care assays of platelet reactivity would demonstrate reduced response to antiplatelet therapy in patients who experienced Drug Eluting Stent (DES) ST whilst on dual antiplatelet therapy compared to matched DES controls. Whilst the aetiology of stent thrombosis (ST) is multifactorial there is increasing evidence from laboratory-based assays that hyporesponsiveness to antiplatelet therapy is a factor in some cases.

**Methods:**

From 3004 PCI patients, seven survivors of DES ST whilst on dual antiplatelet therapy were identified and each matched with two patients without ST. Analysis was performed using (a) short Thrombelastogram PlateletMapping™ (TEG) and (b) VerifyNow Aspirin and P2Y12 assays. TEG analysis was performed using the Area Under the Curve at 15 minutes (AUC15) as previously described.

**Results:**

There were no differences in responses to aspirin. There was significantly greater platelet reactivity on clopidogrel in the ST group using the Accumetrics P2Y12 assay (183 ± 51 vs. 108 ± 31, p = 0.02) and a trend towards greater reactivity using TEG AUC15 (910 ± 328 vs. 618 ± 129, p = 0.07). 57% of the ST group by TEG and 43% of the ST cases by Accumetrics PRU had results > two standard deviations above the expected mean in the control group.

**Conclusion:**

This study demonstrates reduced platelet response to clopidogrel in some patients with DES ST compared to matched controls. The availability of point-of-care assays that can detect these responses raises the possibility of prospectively identifying DES patients at risk of ST and manipulating their subsequent risk.

## Background

Robust evidence demonstrating the ability of drug-eluting stent (DES) technology to reduce restenosis in comparison to bare metal stents (BMS) has led to widespread DES uptake. Balanced against this significant clinical benefit, however, is concern about the incidence of stent thrombosis (ST) in DES patients. Observational and randomised trial data suggest that there is a cumulative incidence of ST in DES patients of between 0.5%–1% per year [1,2], correlating in some series with a similar rate of death and myocardial infarction [3].

There are well established procedural risk factors for ST such as stent under-deployment [4], length of stented segment [5], and idiosyncratic factors including a form of hypersensitivity [6]. However, the inappropriate termination of aspirin and clopidogrel therapy appears to be particularly hazardous [7]. In DES patients there is therefore an important reliance for some (as yet undetermined) period of time on dual antiplatelet therapy [8,9], possibly as a result delayed stent endothelialisation [10]. This requirement for ongoing dual antiplatelet therapy, together with evidence of considerable biological variability in the response of individuals to antiplatelet therapies, particularly clopidogrel [11,12], and association between poor response and adverse cardiovascular outcome [13,14] has raised the important question: can poor responses to these agents render some individuals at risk of DES ST? There is now growing evidence from laboratory based assays that variability in the response to antiplatelet agents, particularly clopidogrel, can contribute to ST in DES patients [15-21].

Typically, clopidogrel is given in standard doses to patients receiving DES, despite both experimental and clinical data demonstrating important biological variation in response. However, clinical detection of reduced responsiveness to clopidogrel and/or aspirin has been hampered both by a lack of point-of-care assays and by an appropriate definition of what constitutes "resistance" [22].

The purpose of this study was to test the hypothesis that platelet reactivity whilst on aspirin and clopidogrel, assessed using two near patient assays, a novel modification of Thrombelastograph^® ^(TEG) PlateletMapping™ [23] and Accumetrics VerifyNow™, would be significantly greater in a consecutive group of patients who survived DES ST than in matched DES controls.

## Methods

### Study Population

Approval was obtained from the Isle of Wight, Portsmouth & South East Hampshire Research Ethics committee prior to commencing the study (Ref: 06/Q1701/49). All subjects provided written informed consent.

Twenty-two patients with DES ST were identified from a consecutive series of 3004 patients, 90% of whom received DES, over a 2 year period at this centre [24]. Seven cases (four subacute and three late) were identified where ST occurred in the context of dual antiplatelet therapy with both aspirin and clopidogrel and in whom dual antiplatelet therapy was on-going. None of the cases were taking additional antiplatelet therapy, anticoagulants or non-steroidal anti inflammatory medication.

For each case two control patients were identified from the interventional database, and individually matched according to duration and dose of antiplatelet therapy, gender, age, smoking, diabetes, initial presentation and procedure undertaken. A summary of the demographics and baseline haematological variables of the cases and controls is given in Tables 1 and 2.

**Table 1:** Demographics

	ST cases	Individual Controls	p value
Age	61.9 ± 5.7	61.8 ± 4.3	N/S
Sex (% Male)	86	86	N/S
Smokers (%)	42.9	35.7	N/S
Diabetes (%)	14.3	14.3	N/S
Emergency cases (%)	14.3	14.3	N/S
Elective cases (%)	14.3	33.3	N/S
ACS cases (%)	71.4	52.4	N/S
Stent length (mm)	26.7 ± 11.3	27.9 ± 6.3	N/S
Minimum stent diameter (mm)	2.8 ± 0.3	2.9 ± 0.3	N/S
Aspirin dose (mg)	140 ± 21.0	140 ± 13.4	N/S
Clopidogrel dose (mg)	86 ± 21	75	N/S
Duration of clopidogrel (days)	161 ± 163	77 ± 28	N/S
Time from latest event/intervention (days)	145 ± 84	76 ± 28	N/S
Statin (% total/% Atorvastatin)	100/14	100/7	N/S

Summary of demographics in the seven cases and 14 controls. There are no significant differences between the groups.

**Table 2:** Haematological variables

	ST cases	Controls	p value
Hb conc. (g/l)	130 ± 13	137 ± 10	0.30
Haematocrit (%)	0.39 ± 0.03	0.40 ± 0.03	0.44
Platelet count	262 ± 42	229 ± 25	0.35
INR	1.1 ± 0.1	1.0 ± 0.0	0.15
eGFR (mls/min)	67 ± 8	64 ± 4	0.64

Haematological variables. There were no significant differences between the two groups.

### Venesection

In all subjects venesection was performed from the antecubital fossa using an 18 gauge needle and a lightly applied tourniquet and the first 2 mls of blood discarded. Blood was then drawn into a 6 ml Lithium Heparin Vacutainer^® ^for TEG analysis and two 2 ml 3.2% sodium citrate Vacutainers^® ^for VerifyNow analysis.

### Sample analysis

#### TEG

samples were analysed using TEG PlateletMapping™ (Haemoscope Corp, IL, USA) according to the manufacturer's instructions. For each trace the maximum amplitude (MA) was obtained from the standard analysis package and the Area under the response curve at 15 minutes (AUC15) calculated using a software programme (Areafinder 2:1) and method of analysis developed and previously described by this group [23,25].

#### VerifyNow

VerifyNow™ Aspirin and P2Y12 assays were analysed for each individual according to manufacturer's instructions to obtain the aspirin response units (ARU) and platelet response units (PRU) respectively.

### Result analysis

For TEG samples comparisons were made between the MA and AUC15 for each individual channel in the 2 groups. The percentage platelet inhibition (%PIn) and percentage clotting inhibition (%CIn) were calculated for both aspirin (using the channel with Arachidonic Acid (AA) stimulation) and clopidogrel (using Adenosine DiPhosphate (ADP) stimulation) from each sample [23,26]. For Accumetrics comparisons were made between the ARU and PRU.

### Statistics

Expert statistical advice was obtained prior to the commencement of the study. Data are presented as the mean and 95% confidence interval of the mean. Significance between the groups was determined using two group t-tests for continuous variables and χ^2 ^(chi squared) tests for categorical data with a p value of < 0.05 (2-tailed) considered to represent significance.

## Results

Baseline demographics are shown in Table 1. There were no significant differences between the 2 groups which were well matched, nor were there any significant differences in baseline haematological variables between the 2 groups (Table 2).

### Responses to aspirin

#### TEG

There was no significant difference in responses assessed with AA-stimulation between the ST group and control group in (i) %PIn (79.6 ± 20.8 vs. 89.9 ± 7.6, p = 0.39); %CIn (75.2 ± 9.4 vs. 75.9 ± 6.0, p = 0.90); (ii) MA of the AA channel (25.3 ± 17.6 vs. 14.0 ± 4.5, p = 0.26) or (iii) AUC15 of the AA channel (244 ± 113 vs. 216 ± 56, p = 0.67).

#### VerifyNow

There was no significant difference between ARU measurements in the ST group and controls (453 ± 50 vs 410 ± 29, p = 0.18).

### Responses to Clopidogrel

#### TEG

**Figure 1:**
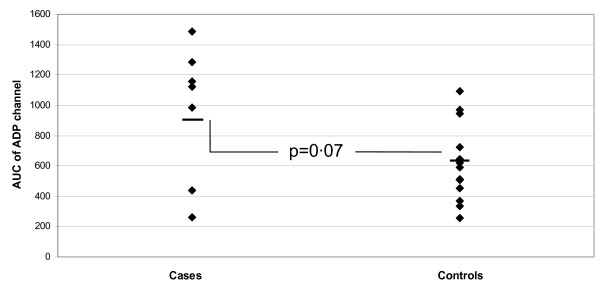
**TEG Results**. The AUC15 of the ADP channel in the ST group and in matched controls. Whilst there was no significant difference between the two groups (910 ± 329 vs. 618 ± 130, p = 0.07) Four of the five greatest responses (signifying the least response to clopidogrel) occurred in the ST group. {AUC15 – area under the response curve at 15 minutes; ADP – Adenosine Diphosphate; ST – Stent thrombosis}.

There was no significant difference between the ST group and controls in the %PIn due to Clopidogrel (18.2 ± 33.2 vs. 31.8 ± 13.2, p = 0.38); the %CIn (5.6 ± 27.7 vs. 30.6 ± 13.4, p = 0.09 or the MA of the ADP channel (50.5 ± 15.8 vs. 46.0 ± 7.5, p = 0.62). There was trend towards significance with the AUC of the ADP channel (910 ± 329 vs. 618 ± 130, p = 0.07) with 57% (4 of 7) of the ST group compared to 0% of controls having an AUC > 1100 (AUC > 2 standard deviations above the mean of the control group) (See Figure 1).

#### Other TEG variables

There were no significant differences between cases and controls in TEG variables which have been previously identified as predictive of ischaemic events after PCI (i.e. MA of the Thrombin channel (61.6 ± 2.9 vs. 64.1 ± 2.7, p = 0.21) and the R time of the Thrombin channel (6.9 ± 1.5 vs. 7.1 ± 2.0, p = 0.84)) [14].

#### VerifyNow

**Figure 2:**
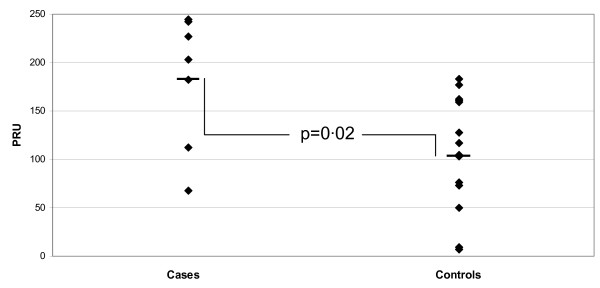
**Accumetrics Results**. VerifyNow PRU in the ST group and in matched controls. Mean PRU was significantly higher in the ST group than in matched controls ((183 ± 51 vs. 108 ± 31, p = 0.02)). The five greatest responses (signifying the least response to clopidogrel) all occurred in the ST group. {PRU – Platelet Reaction Units; ST – Stent Thrombosis}.

The PRU in the ST group was significantly higher than in controls (183 ± 51 vs. 108 ± 31, p = 0.02). 43% (3 of 7) of the ST cases compared to 0% of controls had PRU > 225 (PRU > 2 standard deviations above the mean of the control group) (See Figure 2).

## Discussion

The hypothesis for this study was that these two point-of-care tests (VerifyNow and a novel modification of TEG that produces a result after only 15 minutes) could detect reduced responsiveness to antiplatelet therapy in survivors of ST whilst on dual antiplatelet therapy compared to matched controls. This hypothesis has been disproved in the case of aspirin but proved for clopidogrel using Accumetrics with a trend towards significance using TEG AUC15. Our results suggest that clopidogrel, but not aspirin, resistance contributes to ST in some cases.

### Implications

The implications of this study are clinically relevant. Firstly, they add to the growing concern that some patients receiving DES may be intrinsically at risk of ST because of their relative lack of response to clopidogrel. Secondly, there are now two point-of-care assays available that allow rapid detection of the response of prospective DES patients to clopidogrel, raising the possibility that such "at risk" individuals could be detected in routine clinical practice, before they are exposed to the potentially suboptimal combination of DES and standard doses of antiplatelet drugs. We found that approximately 50% of patients with previous ST whilst on clopidogrel had abnormally high reactivity whilst on clopidogrel with both Accumetrics and TEG (PRU and TEG AUC15 of ADP channel > 2 standard deviations above the expected means). Further data are now required to determine (a) whether the responses of such individuals to antiplatelet therapy can be normalised by interventions such as increasing the maintenance dose of clopidogrel [27], or through the use of alternative or additional antiplatelet agents [28,29], and (b) whether prospectively screening large populations of patients undergoing percutaneous intervention using such tests to guide therapy can decrease ST rates.

### Study limitations

This study has important limitations. Firstly, the absolute number of ST patients is low. Nevertheless, the ST population in this study was derived from a consecutive series of 3004 DES patients at a single centre. Secondly, as this is a retrospective study we do not have baseline samples prior to the initiation of antiplatelet therapy for calculation of a response to antiplatelet therapy compared to baseline. Instead we utilised the clinically important measure of platelet reactivity whilst on antiplatelet treatment. Thirdly, studies recruiting patients who have survived ST will inevitably be weaker for the absence of data relating to patients who died as a result of ST. Lastly we did not test for evidence of drug compliance in this study.

## Conclusion

Using assays suitable for point-of-care use in routine clinical practice, this study demonstrates an association between reduced responses to clopidogrel, but not aspirin, in a proportion of DES ST patients when compared to matched DES patients without ST. These findings are in keeping with recent studies using laboratory based research tools. The availability of two point-of-care assays that can be employed to detect such responses raises the possibility of detecting clopidogrel hypo-responsiveness prior to DES implantation and possibly manipulating the risk of subsequent ST. Further data are required.

## Competing interests

Dr KD Dawkins is on advisory boards for Conor Medsystems, Nycomed, Medtronic, Boston Scientific Corporation and Abbott Vascular and has received honorarium from Boston Scientific Corporation. Dr Curzen has acted as a consultant for and/or received unrestricted research grants from Cordis, Boston Scientific Corporation, Lilly, Smith Klein Beecham, Medtronic, Nycomed and Pfizer International.

## Authors' contributions

AH, KD and NC participated in study design. AH and GM participated in recruitment and data acquisition. AH and GP participated in result analysis. AH and NC drafted the manuscript. All authors have participated in preparation of the final manuscript. All authors have read and approved the final manuscript.
